# Background check: cross-cultural differences in the spatial context of comic scenes

**DOI:** 10.1515/mc-2023-0027

**Published:** 2023-11-01

**Authors:** Fred Atilla, Bien Klomberg, Bruno Cardoso, Neil Cohn

**Affiliations:** Department of Cognitive Science and Artificial Intelligence, Tilburg University, Tilburg, The Netherlands; Department of Communication and Cognition, Tilburg University, Tilburg, The Netherlands

**Keywords:** comics, spatial information, visual language, background, context, cultural differences

## Abstract

Cognitive research points towards cultural differences in the way people perceive and express scenes. Whereas people from Western cultures focus more on focal objects, those from East Asia have been shown to focus on the surrounding context. This paper examines whether these cultural differences are expressed in complex multimodal media such as comics. We compared annotated panels across comics from six countries to examine how backgrounds convey contextual information of scenes in explicit or implicit ways. Compared to Western comics from the United States and Spain, East Asian comics from Japan and China expressed the context of scenes more implicitly. In addition, Nigerian comics moderately emulated American comics in background use, while Russian comics emulated Japanese manga, consistent with their visual styles. The six countries grouped together based on whether they employed more explicit strategies such as detailed, depicted backgrounds, or implicit strategies such as leaving the background empty. These cultural differences in background use can be attributed to both cognitive patterns of attention and comics’ graphic styles. Altogether, this study provides support for cultural differences in attention manifesting in visual narratives, and elucidates how spatial relationships are depicted in visual narratives across cultures.

## 1 Introduction

Research on human cognition has shown cultural variation in the way humans perceive scenes and environments, i.e., scene perception (Masuda 2017; Võ 2021). Whereas people from North America and Europe fixate more on focal objects in scenes, those from Asia focus more on the surrounding scene in the background (Chua et al. 2005; Masuda 2017). Similarly, while people from Western countries focus more on salient objects, those from East Asia focus more on the relationship between these objects and background elements (Boduroglu et al. 2009), and describe events with more references to the context than people from the United States (Masuda and Nisbett 2001). Consequently, these patterns of visual attention can shape esthetic preferences and artistic expression in cultural products; tangible representations of a culture’s dominant meaning system (Masuda 2017; Senzaki et al. 2014; Wang et al. 2012). For instance, East Asian paintings, drawings, and pictures depict more contextual information such as trees and clouds, while Western paintings and pictures further emphasize faces and figures (Masuda et al. 2008). In addition, aesthetically, people from the United States prefer pictures with small backgrounds and large figures, while those from East Asia prefer the opposite (Masuda et al. 2008). This extant research has thus suggested that people from Western cultures are more object-oriented with visual scenes, while those from East Asia are more context-oriented.

While cognitive studies have mainly examined cultural differences in standalone visual scenes such as single drawings and photographs, such differences may also extend to complex sequential scenes in visual narratives such as comics (Cohn 2020). In comics, scenes are typically presented as units of “panels”, with focal characters and objects in the foreground, and contextual information about environments in the background. Yet, because of their sequential nature, spatial and environmental information is not only conveyed by individual panels but also constructed and shifted across sequences of images. This interplay between individual panels and their sequence therefore makes visual narratives such as comics a promising medium to examine cognitive and linguistic processes, such as the construction of space through graphics. This is evident from the variety of approaches to visual narrative comprehension and creation, with theories based on psycholinguistics (Cohn 2020), multimodal discourse (Bateman and Wildfeuer 2014), scene perception (Loschky et al. 2020), and even computational modeling (Chen and Jhala 2021; Martens et al. 2020).

Some of these more cognitive approaches have already incorporated cultural differences in visual narrative processing (Cohn 2020; Loschky et al. 2020). Similar to research on visual attention, visual narrative research began with comparisons between American comics and Japanese manga, as these are popular comics originating from a Western and Asian culture, respectively, and both have spread to have worldwide influence. American comics and Japanese manga differ in several dimensions. For instance, mainstream American comics are often published in full color, while Japanese comics are generally published in black and white. American comics are read from left to right, while Japanese comics are read from right to left, and they differ in the properties of their layouts (Cohn et al. 2019), their storytelling (Klomberg et al. 2022), and their perspective taking (Cohn et al. 2022). These and other differences have suggested that comics do not use universal structures but are created using distinctive “visual languages” that may differ across cultures (Cohn 2018, 2024).

Comics also differ cross-culturally in how they convey information in scenes, aligning with cultural patterns of visual attention. Several studies have now shown that American and Japanese comics frame aspects of a scene in different ways (Cohn 2011, 2020, 2024; Cohn et al. 2012). Panels were categorized depending on the number of “active entities”, i.e., characters or objects of primary focus in a scene. American and European comics had more panels depicting multiple active entities, while Japanese and Chinese comics used more panels with one individual active entity, close-ups of an active entity, or panels with no active entities, often depicting only a landscape or environment. A possible explanation for this difference in framing is that Japanese comics tend to break up a larger environment by highlighting smaller, detailed parts of that environment in multiple separate panels (Cohn 2015; Cohn et al. 2012; McCloud 1993). This interpretation would be in line with prior research on cross-cultural attention, as distributing a scene across panels may simulate the way that readers’ eyes fixate on different aspects of a scene, forcing readers to piece the parts together to form the whole environment (Cohn 2011).

Previous studies on framing attention focused on the amount of information in a panel, rather than the spatial representation of the scene. Such contextual information about the location and environment is commonly depicted through the background. Backgrounds have been largely neglected in research on visual narratives, despite constituting a large proportion of space in panels. As such, little is known about how backgrounds convey information about the environment, location, and other spaces a narrative takes place in. This raises questions such as whether backgrounds convey spatial information in alignment with a culture’s attentional orientation as object- or context-oriented. For instance, if Japanese manga truly distribute spatial information across panels, with fewer focal characters or objects in general (Cohn 2015; Cohn et al. 2012; McCloud 1993), would panels retain drawn backgrounds with detailed spatial information when focal characters or objects are present?

Moreover, sequences of panels can construct notions of scenes, as the background in each panel reveals details about the location or environment of the narrative. Therefore, it is also important to understand how backgrounds are used across multiple panels to convey contextual information. In their analysis of scene perception in visual narratives, Loschky et al. (2020) suggest that American comics reduce the need for readers to actively track scenes across panels by first establishing the environment in full, and then leaving out backgrounds entirely in the following panels. In films, research has already found that new environments tend to be shown for the first time with more background information over a longer duration (Cutting and Iricinschi 2015).

Empirical evidence for this pattern in comics has yet to be presented, including whether American comics are unique in this regard. Participants from East Asia have been found to not only attend but also remember more background information than those from the United States (Masuda and Nisbett 2001; Nisbett and Miyamoto 2005). Accordingly, East Asian comics might not need to convey the context or environment of a scene as often as American comics to their readers, consequently conveying less spatial information through background scenery. Addressing these questions requires empirical investigation into how often backgrounds communicate contextual and environmental information *explicitly*, with information that helps readers directly recognize a location within the narrative.

In order to answer the questions raised above, this study examines how comics convey contextual information about the spatial location and environment of a scene, referred to as the *spatial context* in this paper. Specifically, we study (1) how backgrounds are used to convey the spatial context, and (2) how often the spatial context is conveyed explicitly. Furthermore, to examine whether it is truly a culture’s attention orientation that shapes background use, we compared comics from six countries that varied in their crossing of visual attention styles and comic graphic styles. Comics from two East Asian countries, Japan and China, were included due to the similar patterns of visual attention from people in those countries (Masuda et al. 2008; Ueda and Komiya 2012), but use of different graphic styles in their comics. These were further contrasted with ‘manga’ from Russia, which emulated the Japanese manga style, while originating from a distinct culture amidst Western and Asian (Baranovsky 2000; O’Loughlin and Talbot 2005; Varnum et al. 2008). Likewise, we included comics from two Western countries with similar attention orientation, the United States and Spain (San Martin et al. 2019). These were contrasted with Nigerian comics resembling American superhero comics in style, while the visual attention of Nigerians has been shown to be more context-oriented, closer to East Asian countries (San Martin et al. 2019). These contrasts allow us to compare whether background use is related to human attention orientation or comic graphic style.

If comics depict backgrounds in line with cognitive research showing that people from East Asia are more context-oriented than those from Western countries (Masuda 2017), we hypothesize that comics from East Asian countries (Japan and China) will contain more backgrounds that show the spatial context in detail than Western comics (America and Spain). While Russian and Nigerian comics emulate the graphic style of Japan and the United States, respectively, we do not expect them to use backgrounds similarly to comics from the countries they emulate. To this end, we hypothesize that graphic style is a more superficial structure while the expression of attentional orientation connects to more subtle structures of visual narration.

## 2 Methods

### 2.1 Data collection

We collected a sample of 60 comics from the TINTIN Corpus (Supplementary Material 1; www.visuallanguagelab.com/tintin). Comics from six different countries matching the aforementioned graphical styles were chosen through convenience sampling. All comics were provided by online sources, comic creators, publishers, and other collaborators. Our sample analyzed 10 comics each from Japan, China, Russia, Nigeria, Spain, and the United States, together adding up to a total of 6,716 panels across 1,274 pages (Table 1).

**Table 1:** Aggregated information of included comics from each country. For detailed information about each comic, see the full list of titles in Supplementary Material 1.

Country	Total comics	Total pages	Total panels	Panels per page
Japan	10	294	1,644	5.59
China	10	245	1,090	4.45
Russia	10	192	936	4.88
Nigeria	10	148	665	4.49
Spain	10	201	1,346	6.70
United States	10	194	1,035	5.34
Grand total	60	1,274	6,716	5.27

### 2.2 Data annotation

**Figure 1:**
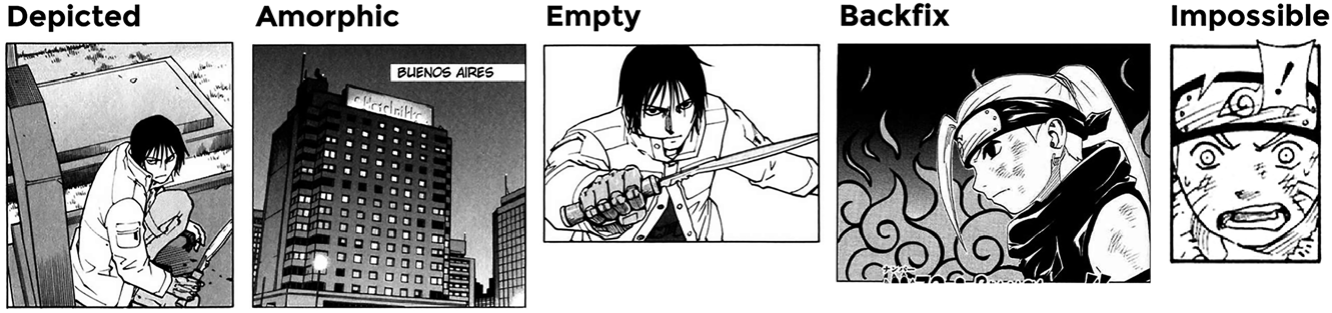
Possible background types within a panel. Panels 1–3 sourced from *Eden: It’s an Endless World!* by Hiroki Endo, panels 4 and 5 from *Naruto* by Masashi Kishimoto.

Each comic was annotated using the Multimodal Annotation Software Tool (MAST, version 0.8; Cardoso and Cohn 2022), which facilitates the annotation of specified regions in multimodal documents. The documents we analyze in this study feature a single region per panel, each annotated both as a panel and according to the type of its depicted background (Figure 1).

A framework of background types was developed based on the explicitness of contextual information conveyed (Klomberg 2022). The most explicit manner of conveying the spatial context we identified was by depicting a recognizable environment or location in the background. Such explicit backgrounds can be accompanied by a (main) character or object in the foreground as the focus of the narrative. These were termed depicted backgrounds. However, backgrounds can also be explicit when no such active entities are present (Cohn et al. 2012), termed amorphic backgrounds. A further distinction was made for panels that show active entities in the foreground but convey no recognizable space from the narrative in the background, hence conveying the spatial context more implicitly. For instance, in Japanese manga, pictorial patterns in the background can be used to convey the emotional state of characters (Cohn and Ehly 2016; Shinohara and Matsunaka 2009), e.g., flowers behind a character in love or thunder behind angry characters. These were called “Backfix” backgrounds, because they are visual “affixes” that are set in the spatial background of the scene. Furthermore, it is possible that no spatial information is drawn at all, but instead, the space around the active entities is left empty or filled with a single color or gradient. We refer to such cases as “Empty” backgrounds.1Empty backgrounds are named ‘No’ backgrounds in the original annotation scheme (Klomberg 2022). Finally, some panels show an object or body part zoomed in so far that it completely fills the panel (Cohn et al. 2012). In these panels, there is no room for any background to be drawn, and thus our framework categorizes these as “Impossible” backgrounds.

### 2.3 Data analysis

Two quantitative dependent variables were operationalized per comic using Python (v3.9). First, to measure the use of backgrounds, we calculated a proportion for each background type by dividing the number of panels containing each background type by the total number of panels in each comic. Second, to assess how often sequences conveyed the context explicitly, we derived the “re-establishing length”. Here, we averaged the number of panels between instances of one explicit background (Amorphic or Depicted) and the next explicit background in the sequence. The lengths of such “gaps” with only implicit backgrounds (Empty, Backfix, Impossible) were additionally explored and plotted.

JASP (v0.16.3) was used to statistically test for differences in each dependent variable within and between countries. To examine how different backgrounds are used to convey the spatial context, mixed model ANOVAs were performed with country as a between-subjects factor and background as a within-subjects factor on background proportions. Follow-up simple effects analyses were performed per country and background type using one-way ANOVAs. To examine how often the spatial context is conveyed explicitly, a one-way ANOVA with country as a between-subjects factor was run on the re-establishing length. Post-hoc tests were performed to compare groups pairwise with the conservative Bonferroni correction applied to adjust the *p*-values for multiple comparisons to reduce the risk of type I errors. A Greenhouse-Geisser correction was used when Mauchly’s test indicated that the assumption of Sphericity was violated. A Welch ANOVA followed by Games-Howell post-hoc tests was employed where Levene’s test indicated the assumption of homogeneity of variances was violated.

## 3 Results

### 3.1 Background use

**Figure 2:**
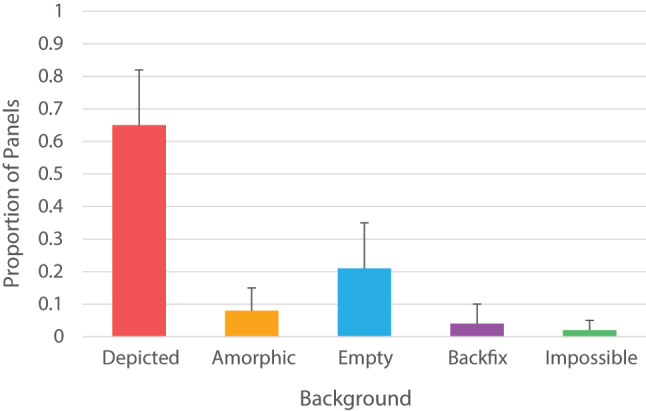
Mean proportion of background types across all 60 comics.

We first analyzed the frequency differences between background types, which differed from each other, *F*(1.82, 98.33) = 416.24, *p* < 0.001, *η*_*p*_^2^ = 0.89. Post-hoc tests showed that depicted backgrounds were used more than every other background type (Figure 2). In addition, empty backgrounds were more frequent than amorphic, backfix, and impossible backgrounds, which in turn did not differ in their proportions. It appears that globally, depicted backgrounds are most frequently used, followed by empty backgrounds. The complete list of post-hoc statistics for the pairwise comparisons can be found in Supplementary Material 2. There was no main effect of country, *F*(5, 54) < 0.01, *p* = 1, *η*_*p*_^2^ < 0.01. This is expected as the ANOVA averages the proportions of all background types and thus each country’s mean background proportion was 0.20.

Most relevant to this study, an interaction arose between country and background type *F*(9.11, 98.33) = 5.81, *p* < 0.001, *η*_*p*_^2^ = 0.35, which was followed up by simple effects analyses for each country and background type. As the number of tests was extensive, only main effects and significant pairwise comparisons are reported, with detailed statistics available in the Appendix.

One-way ANOVAs revealed a simple main effect of background type on proportions for Japanese, Chinese, Russian, Nigerian, Spanish, and American comics (Table 2). Post-hoc tests revealed similar patterns to those mentioned above for the global average (Supplementary Material 2). In all six countries, depicted backgrounds were used more than every other type. Furthermore, Japanese, Russian, Chinese, and American comics used empty backgrounds more than impossible and backfix backgrounds. Japanese and Russian comics also used empty backgrounds more than amorphic backgrounds. In contrast, Nigerian and Spanish comics showed no significant differences between any of these four background types.

**Table 2:** Simple main effect of background type on proportions for each country as shown by a separate RM ANOVA. Mauchly’s test indicated that the assumption of sphericity was violated for each country (*p* < 0.05), hence a Greenhouse-Geisser correction was used.

Country	Sum of squares	df	Mean square	*F*	*p*	*η*_*p*_^2^
Japan	2.024	2.276, 20.487	0.889	98.039	<0.001	0.916
China	2.23	1.565, 14.083	1.425	30.801	<0.001	0.774
Russia	1.944	2.055, 18.495	0.946	66.882	<0.001	0.881
Nigeria	3.202	1.948, 17.536	1.643	93.424	<0.001	0.912
Spain	4.725	1.158, 10.418	4.082	162.689	<0.001	0.948
USA	3.771	1.079, 9.713	3.494	67.659	<0.001	0.883

**Figure 3:**
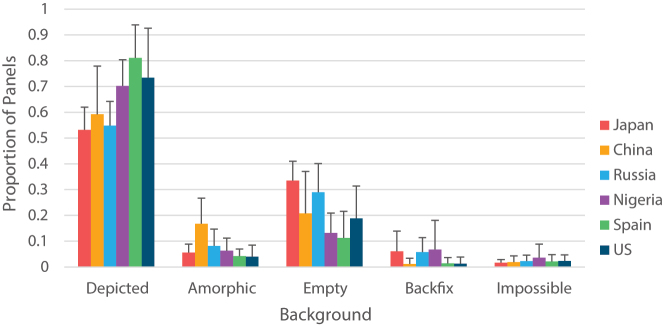
Mean proportion of background types in each country’s comics.

One-way repeated measures ANOVAs also showed significant simple main effects of country on the proportions of depicted, amorphic, and backfix backgrounds (Table 3). As illustrated in Figure 3, Chinese comics used amorphic backgrounds more than Spanish and American comics. Spanish comics used depicted backgrounds more often than Chinese, Japanese, and Russian comics, while American comics only used them more than Japanese. Finally, both Japanese and Russian comics used empty backgrounds more than Nigerian and Spanish comics. No main effect of country appeared for impossible or backfix backgrounds, thus no country comparisons were made.

**Table 3:** Simple main effect of country on each background type’s proportion as shown by a separate one-way ANOVA.

Background type	Homogeneity correction	Sum of squares	df	Mean square	*F*	*p*	*η*_*p*_^2^
Depicted	None	0.633	5, 54	0.127	6.592	<0.001	0.379
Amorphic	Welch	0.116	5, 24.713	0.023	3.3	0.02	0.388
Empty	None	0.379	5, 54	0.076	5.909	<0.001	0.354
Backfix	Welch	0.037	5, 24.525	0.007	2.079	0.102	0.148
Impossible	None	0.002	5, 54	<0.001	0.497	0.777	0.044

### 3.2 Re-establishing length

**Figure 4:**
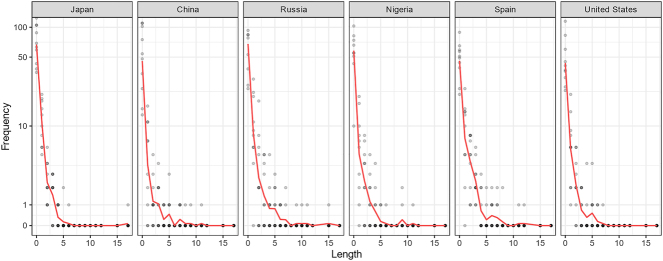
Frequencies (on a log scale *y*-axis) of re-establishing lengths in comics for each country.

The re-establishing length – the average number of panels between those with explicit backgrounds – was explored first. Globally, the 60 comics together had an average re-establishing length of 0.45 panels (SD = 0.36), ranging from 0 at minimum to a maximum average of 1.67 panels. A re-establishing length of 0 indicates there were comics that conveyed the context explicitly in every panel using amorphic or depicted backgrounds, never using any of the three implicit background types. On the other extreme end, one comic solely showed implicit backgrounds for 1.67 panels on average after conveying the spatial context explicitly. Looking at the frequency of all possible re-establishing lengths across the corpus of comics, the most frequent length was 0 (Figure 4).

Figure 4 further shows the frequency of re-establishing lengths for comics from each country. All countries most often had a re-establishing length of 0 in their sequences, followed by gaps of 1, 2, and so forth, successively decreasing in frequency with longer lengths. The frequencies of lengths for each country appear to follow a power law distribution, where a small number of items, in our case lengths, appear most frequently.

**Figure 5:**
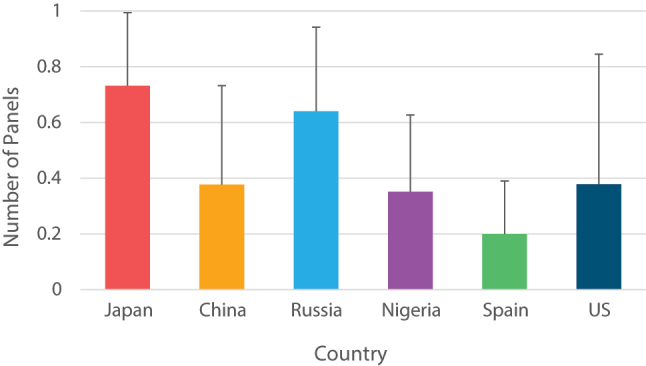
Average number of panels it takes to re-establish the context in comics from each country.

Despite the consistency of these length by frequency relationships across all countries in the sample, comics from varying countries differed in their average re-establishing lengths. We found a main effect of country on the average re-establishing length, *F*(5, 54) = 3.88, *p* = 0.004, *η*_*p*_^2^ = 0.26. Both Japanese and Russian comics took significantly longer than Spanish comics to re-establish the context (Figure 5). No other comparisons differed significantly in their average establishing length (Table 11 in Supplementary Material 2).

## 4 Discussion

This study examined cross-cultural differences in the use of backgrounds to convey the spatial context in scenes through analyses of a corpus of 60 annotated comics from six different countries (10 per country). In line with cognitive research on attention and other cross-cultural studies on comics (Cohn 2018, 2020; Masuda 2017), cross-cultural differences arose in the use of backgrounds to convey the spatial context. Specifically, comics were found to group along their use of either explicit or implicit strategies to convey the spatial context through backgrounds. Comics from the United States, Spain, and Nigeria conveyed the context more often by explicitly depicting recognizable locations and environments in the background. Implicit strategies on the other hand were employed by comics from Japan, China, and Russia, which more often omitted recognizable spaces in the background, leaving it up to readers to infer the spatial context.

Globally, different types of backgrounds were used to varying degrees to convey the spatial context of comic scenes. Comics most often depicted the background in detail behind an active entity to convey the spatial context (i.e., depicted backgrounds), followed by entirely omitting spatial information by showing a single color or gradient behind a character in the foreground (i.e., empty backgrounds). Other types of backgrounds (amorphic, impossible, and backfix) were used less often. This pattern is not unexpected, as depicted and empty backgrounds have active entities in the foreground, which comics are expected to have for most of their panels in order to tell a story (matching Cohn et al.’s (2012) macro or mono framing). The remaining three backgrounds could then be used in more targeted ways: amorphic backgrounds to purely convey contextual and environmental information as the focus, aligning with prior findings about amorphic framing (Cohn 2020; Cohn et al. 2012), impossible backgrounds to fully focus on “micro” framing of an active entity, and lastly, backfixes to convey the emotions and mood of a scene.

Looking at background use in comics from specific countries, Japanese, Chinese, Russian, Nigerian, Spanish, and American comics all followed the global pattern by using depicted backgrounds most often. Empty backgrounds were in turn used second most often in all countries except Spain and Nigeria. However, whereas American and Chinese comics used empty backgrounds more often than impossible and backfix backgrounds, manga-style comics from Japan and Russia also used them more often than amorphic backgrounds. This suggests comics from around the world differ in their use of specific types of backgrounds. This was further confirmed by the subsequent cross-country comparisons we performed, to be discussed next.

Specifically, the results align with cross-cultural differences in the use of three types of backgrounds. Manga-style comics from Japan and Russia implied the context using empty backgrounds more often than comics from Nigeria and Spain. Chinese comics used amorphic backgrounds, entire panels filled with environments without any focal characters or objects, more often than Spanish and American comics. In contrast, Spanish and American comics depicted the background more frequently with a focal character or object in the foreground than Japanese comics. Spanish comics additionally used such backgrounds more than Chinese and Russian comics. While depicted backgrounds are drawn in a way that conveys the context explicitly, empty backgrounds can only convey the context implicitly. Taken together, these findings indicate that Japanese and Russian comics convey the spatial context less often than American, Nigerian, and Spanish comics. Distinctly, Chinese comics convey the context as explicitly as Western comics, but leave out active entities, as characterized by their use of amorphic backgrounds.

Initially, we hypothesized that Japanese manga would convey the spatial context more explicitly due to their context-oriented attention (Masuda 2017). However, given the results, our reasoning might have been inverted. Perhaps, conveying the context too explicitly could be distracting for Japanese readers, as they would constantly allocate attention to the background. This explanation can be further supported by cognitive studies on attention: people from East Asia not only tend to attend more to the context but also appear to find it more difficult to separate an object from the field it appears in (Nisbett and Masuda 2003). Therefore, Japanese comics might leave the background empty as a “break” from all the detailed spatial information conveyed by depicted and amorphic backgrounds. Indeed, even in historical works dating back to the early 20th century, Japanese manga creators were said to prefer fewer explicitly drawn backgrounds than Americans (Exner 2021). As a result, manga can more carefully organize whether the focus of a panel is on the environment or on active entities (Cohn et al. 2012; McCloud 1993). In contrast, the use of depicted backgrounds in American and Spanish comics would require readers to selectively attend to focal objects and characters in a scene without exhausting their attentional resources on background information.

These broader differences related to spatial explicitness are further supported by the length it took to re-establish the spatial context. As only depicted and amorphic backgrounds explicitly convey the spatial context, we looked at the distance between instances of these panels. Here again, comics from different countries differed in their re-establishing length in a way suggesting differences between the preferred explicitness of spatial context. In line with the lower frequency of explicit backgrounds, Japanese and Russian comics took longer to re-establish the context than Spanish comics. Thus, relative to comics from a Western country, manga-style comics have longer sequences where the spatial context is not conveyed explicitly.

Across comics from different countries, the length it took to re-establish the spatial context appears to be distributed according to Zipf’s Law, a well-known power law where the frequency declines sharply for less common items (Piantadosi 2014). In written language, this pattern was initially observed in English texts where few words, such as *the* and *that*, appeared proportionally most often (Zipf 1949). Furthermore, our distributions also follow the Brevity Law, where the frequency declines sharply for items of longer length, e.g., longer words in linguistics (Corral and Serra 2020). These statistical patterns occur universally in many human communication systems such as verbal language and music, supposedly reflecting a preference of both speakers and listeners to minimize communicative efforts (Piantadosi 2014). Our results suggest that such patterns of conveying information extend to visual language, where authors and readers minimize the effort to communicate spatial information. Indeed, similar trade-offs in the frequency of sequential lengths have been shown in a prior corpus of comics for sequencing of framing types, situational coherence, and the size of rows (Cohn 2024).

Due to the sequential nature of comics, information is repeated in multiple panels to convey the continuity of characters and environments (Klomberg et al. 2022). Loschky et al. (2020) speculated that American comics might reduce the need for readers to actively track scenes across panels by leaving out backgrounds after establishing the environment early on. We found this to be the case for all countries to some extent, as approximately after every two explicit panels there was one implicit panel. Nevertheless, Japanese and Russian comics were most extreme in this regard, with the greatest distance separating panels with explicit spatial information on average, consequently forcing their readers to infer the context the most.

Such inference might be easier on readers of manga-style comics than constantly being exposed to the spatial context. That is, readers familiar with manga-style comics are already habituated to context inference, and thereby can actively track spatial information across panels with more ease. Cognitive research has shown that not only do people from East Asia tend to attend more to background information in visual scenes than those from North America, but they also remember more about it (Masuda and Nisbett 2001; Nisbett and Miyamoto 2005). The idea here would be that by reducing how often a comic conveys the spatial context, less information would need to be tracked across panels by readers, thereby reducing their memory load. With this, readers would assume spatial continuity across panels (Klomberg et al. 2022), needing only to detect important shifts in the spatial context, such as when the narrative moves to another location. More research is required in the future to reveal how the spatial context continues and shifts across longer sequences of panels.

Overall, the cross-cultural observations suggest two distinguishable groupings related to the conveying of spatial context: comics from Japan, China, and Russia, which employ more implicit strategies to convey the spatial context, and comics from the United States, Spain, and Nigeria, which employ more explicit strategies. While not homogenous, comics from the six countries never differed in their background use within their own cultural type. Of note, Spanish and American comics most often contrasted together from the “implicit” comics, while Nigerian comics did not. As participants from Spain and America have been shown to have a similar orientation of attention (San Martin et al. 2019), but differing graphic styles, and participants from Nigeria and America have the opposite pattern of attention but similar styles of their comics, this suggests that background use in comics is related more to visual attention than graphic style.

However, the more implicit comic grouping contradicts this interpretation. Although they came from different cultural origins, the Russian manga were imitative of the visual language originating from Japanese manga, grouping together more often than the culturally similar Chinese and Japanese comics, which have similar context-oriented attention. Is background use in comics then decided more by a country’s attention orientation or rather the unique style of the comics? Previous studies on other comic properties have suggested a mix of both playing a role. For instance, Original English Language manga are comics in the manga style of Japan, made in the object-oriented United States. Recent studies have shown these comics to fall between American comics and Japanese manga in terms of attentional framing and motion events (Cohn 2020; Hacımusaoğlu and Cohn 2022). Our findings extend this role of both attention orientation and graphic style to background use. In this study then, the attention to focal objects in American and Spanish comics could dictate background use first, resulting in the two countries using backgrounds in more similar ways than those from America and Nigeria, which share a superhero style. Similarly, in Russian manga, the background use would be dictated by the imitation of Japanese Visual Language, reducing the influence of their culture’s attention orientation.

Overall, these findings should be interpreted with caution given the size and scope of the sample, but future work can further examine these relationships across a broader cultural scope. Expansion of the TINTIN Corpus is currently underway with comics from over 140 countries, allowing us to extend our analysis to other cultures and other aspects of comics. Additional questions persist about the relationship between these corpus findings and the actual comprehension of visual narrative sequences. Most theories of the processing of visual narratives maintain that visual sequences are understood by readers using processing mechanisms assumed to be universal (Cohn 2020; Loschky et al. 2020). Yet, this work and other corpus analyses suggest that visual narratives differ in their structures, which raises questions about how such differences may interact with their processing. For instance, does the amount of (spatial) inference required by readers increase their cognitive load and subsequently impact their ability to construct a comprehensible mental representation of the drawn space? In answering such questions, future studies can further consider cultural identity, as a country’s culture is not completely homogenous and each person adheres to cultural norms, values, and ideas differently (Matsumoto et al. 1996).

In sum, this study investigated how comics from different countries convey contextual and environmental information within scenes of comics using backgrounds. We found that globally, comics convey the spatial context of scenes most often explicitly, with recognizable environments drawn around the focal characters and objects. However, as has been shown for other structural properties of the visual languages used in comics (Cohn 2018, 2020), background use is not universal. Across countries, we found that comics from Japan, China, and Russia convey the spatial context less often in detail compared to comics from the United States, Spain, and Nigeria. While the latter three countries use more explicit strategies such as frequent, detailed backgrounds with recognizable environments, the former three countries employ implicit strategies such as leaving the background empty of any spatial information. These differences can be attributed to a mix of both cognitive patterns of attention and graphic style. All in all, this work shows that visual narratives provide an informative place to study various aspects of cognition, including our construction of space.

## Supplementary Material

Supplementary Material

Supplementary Material
