# Supplementary material for: Background check: cross-cultural differences in the spatial context of comic scenes
Source: Multimodal Commun. 2023 Nov 1;12(3):179–89. doi: 10.1515/mc-2023-0027 (PMC10740350; doi:10.1515/mc-2023-0027)
Supplement: Supplementary file 1 — Supplementary Material [file j_mc-2023-0027_suppl_001.pdf]

Supplement 1 to *Background Check: Cross-Cultural Differences in the Spatial Context of Comic Scenes*

Comic Title	Authors & Illustrators	Country	Year	Pages	Panels
Berserk	Kentaro Miura	Japan	1993	34	168
Eden: It's an Endless World!	Hiroki Endo	Japan	2007	29	118
Full Metal Panic!	Shoji Gatoh	Japan	1998	30	157
Fullmetal Alchemist	Hiromu Arakawa	Japan	2007	30	191
Kagerou Nostalgia	Satomi Kubo	Japan	1999	24	205
Kaikisen	Satoshi Kon	Japan	1990	28	169
Naruto	Masashi Kishimoto	Japan	2001	36	234
Ranma 1/2	Rumiko Takahashi	Japan	1988	31	139
REAL	Inoue Takehiko	Japan	2003	29	152
Skyhigh: Karma	Tsutomu Takahashi	Japan	2003	23	111
14 days in the desert	Liu Tuo, Xu Zi Ran, Senyu Studio	China	2021	27	126
A Restaurant Nearby	Yao Ren	China	2014	27	132
Cuisine Chinoise	Zao Dao	China	2018	27	125
Echo in Hai Men	Seven	China	2020	29	150
Evolution: The Epic of Earth	Lv Bo	China	NA	20	67
Fly: The Song of frogs	Zhang Xiao Yu	China	2014	22	107
Legend of Tianma	Chu Mi	China	2014	25	94
Night Bus	Zuo Ma	China	2018	20	97
Raven Forest	Lu Yizhou, Amber Ma	China	2020	17	72
The Night of Ghosts	Qian Yu	China	2010	31	120
Anime Nyash	Alexey Kuryatnikov	Russia	2016	17	61
Tagar	Anna Sergeeva, Marina Privalova	Russia	2018	26	130
First One	Amito Arai	Russia	2018	19	86
Inspiration	Dzikawa	Russia	2015	25	99
Once upon a tale	Sideburn004	Russia	2018	18	71
Path to the Future paved with memoria gems	Hetiru, Semkul	Russia	2016	21	129
Pirozhki (Grandmother's pies)	Sideburn004	Russia	2018	9	54
Princess Frog	Ksenia M Belka	Russia	2018	28	170
The Gift of Goddess Ai	Lina & Yu	Russia	2018	9	55
Yakutia	Bogdan Fedotov	Russia	2016	20	81
Amazing Tek Kids	Alexander “Rudeworks” Ighoja, Peter Daniel	Nigeria	2015	13	61
Avonome - The Realm Within	Xavier Ighorodje, Stanley Stanch Obende	Nigeria	2015	11	36
Black Sage - The Rising	Bill Bidiaque, Sola Adebayo	Nigeria	2015	9	44
Chayoma (Curse of the Jangura)	Peter Chizoba Daniel, Isaiah Ovie Gibson, Jimmy King, Ape Ekene Polycap	Nigeria	2020	15	88
Chronicles of the Newborn - Rise of Mlezi	Adeniji Jr, Peter Daniel	Nigeria	2017	23	116
Di Iche - Naija Anomaly	Peter Chizoba Daniel, Nwankwor Newman	Nigeria	2018	18	79
Eru (Pestillence of the Night)	Tobe "Max" Ezeogu, Ozo Ezeogu	Nigeria	2015	7	29
Hero Kekere (Prime Edishun)	Cassandra Mark, Kelechi Isaac	Nigeria	2018	16	63

ShowDown - Chaos Rising	Alexander “Rudeworks” Ighoja	Nigeria	2018	22	96
Tatashe	Cassandra Mark, Tobe Max Ezeogu	Nigeria	2018	14	53
1714 Baluarte	Cels Piñol, Àlex Santaló	Spain	2014	20	111
Ardalén	Miguelanxo Prado	Spain	2012	22	111
Caminantes	Pedro Lobato	Spain	2013	20	58
El arte de volar	Antonio Altarriba	Spain	2009	24	162
El Capitán Coraje 11 El fin de un esbirro	G. Iranzo	Spain	1947	8	78
El Faro	Paco Roca	Spain	2004	23	169
El violeta	Juan Sepúlveda Sanchis, Antonio Mercero	Spain	2018	17	138
Islamundo – Primera temporada	David Pérez Gutiérrez, Jesús Daniel Fernández	Spain	2015	20	65
Ken Games: Piedra	José Robledo, Marical Toledano	Spain	2009	27	271
La casa de los susurros	David Muñoz, Tirso Cons	Spain	2011	20	183
Danger Girl	Jeff Campbell	United States	1997	21	120
Doc Savage	Doug Moench, John Buscema, & Tony DeZuniga	United States	1975	24	146
Emperor Doom	David Michelinie, Bob Hall	United States	1987	20	120
Savage Dragon	Erik Larsen	United States	2014	20	101
Spawn	Todd McFarlane	United States	1992	21	117
Star Wars Annual	Chris Claremont, Mike Vosburs, & Steve Leialola	United States	1979	33	181
Strange Adventures: The Invisible Space-Dog	Gil Kane	United States	1961	8	46
Strange Adventures: The Toy Soldier War	Carmine Infantino	United States	1961	8	36
Strange Adventures: War with the Giant Frogs	Gardner Fox, Sid Greene	United States	1961	8	46
Way of the Rat	Chuck Dixon, Jeff Johnson	United States	2002	31	122
