# Supplementary material for: Background check: cross-cultural differences in the spatial context of comic scenes
Source: Multimodal Commun. 2023 Nov 1;12(3):179–89. doi: 10.1515/mc-2023-0027 (PMC10740350; doi:10.1515/mc-2023-0027)
Supplement: Supplementary file 2 — Supplementary Material [file j_mc-2023-0027_suppl_002.pdf]

## Supplement 2 to *Background Check: Cross-Cultural Differences in the Spatial Context of Comic Scenes*

This material contains the full statistics from post-hoc tests performed for the (simple) main effects of Country or Background type on background proportions (Table 1 to 10) and re-establishing length (Table 11), presented as a matrix with significant comparisons highlighted.

Table 1. *Pairwise comparisons of global background proportions.*

| **Country** | **Mean** | **SD** | **t-statistic, p-value** | | | |
|---|---|---|---|---|---|---|
| | | | **Depicted** | **Amorphic** | **Empty** | **Backfix** |
| **Depicted** | 0.653 | 0.168 | | | | |
| **Amorphic** | 0.075 | 0.071 | 31.594, < .001 | | | |
| **Empty** | 0.211 | 0.135 | 24.163, < .001 | -7.43, < .001 | | |
| **Backfix** | 0.037 | 0.065 | 33.648, < .001 | 2.054, 0.412 | 9.484, < .001 | |
| **Impossible** | 0.023 | 0.029 | 34.423, < .001 | 2.829, 0.051 | 10.26, < .001 | -0.775, 1 |

Table 2. *Pairwise country comparisons for Depicted background proportions.*

| **Country** | **Mean** | **SD** | **t-statistic, p-value** | | | | |
|---|---|---|---|---|---|---|---|
| | | | **Japan** | **China** | **Russia** | **Nigeria** | **Spain** |
| **Japan** | 0.532 | 0.088 | | | | | |
| **China** | 0.592 | 0.187 | 0.968,1 | | | | |
| **Russia** | 0.548 | 0.094 | -0.250, 1 | 0.718, 1 | | | |
| **Nigeria** | 0.702 | 0.102 | -2.742, 0.124 | -1.774, 1 | 2.492, 0.237 | | |
| **Spain** | 0.811 | 0.128 | -4.496, <.001 | -3.528, 0.013 | -4.246, 0.001 | -1.754, 1 | |
| **USA** | 0.734 | 0.192 | -3.260, 0.029 | -2.293, 0.387 | -3.010, 0.059 | -0.519, 1 | 1.235, 1 |

Table 3. *Pairwise country comparisons for Amorphic background proportions.*

| **Country** | **Mean** | **SD** | **t-statistic, p-value** | | | | |
|---|---|---|---|---|---|---|---|
| | | | **Japan** | **China** | **Russia** | **Nigeria** | **Spain** |
| **Japan** | 0.056 | 0.033 | | | | | |
| **China** | 0.168 | 0.099 | 3.405, 0.050 | | | | |
| **Russia** | 0.082 | 0.065 | 1.127, 0.862 | 2.317, 0.245 | | | |
| **Nigeria** | 0.063 | 0.049 | -0.362, 0.999 | 3.027, 0.082 | -0.745, 0.973 | | |
| **Spain** | 0.042 | 0.028 | 0.997, 0.913 | 3.878, 0.025 | 1.780, 0.511 | 1.149, 0.853 | |
| **USA** | 0.04 | 0.045 | 0.894, 0.943 | 3.732, 0.025 | 1.677, 0.564 | 1.076, 0.885 | 0.126, 1 |

Table 4. *Pairwise country comparisons for Empty background proportions.*

| Country | Mean | SD | t-statistic, p-value | | | | |
|---|---|---|---|---|---|---|---|
| | | | Japan | China | Russia | Nigeria | Spain |
| **Japan** | 0.335 | 0.075 | | | | | |
| **China** | 0.208 | 0.163 | -2.496, 0.235 | | | | |
| **Russia** | 0.29 | 0.111 | 0.892, 1 | -1.604, 1 | | | |
| **Nigeria** | 0.132 | 0.077 | 4.002, 0.003 | 1.505, 1 | -3.109, 0.045 | | |
| **Spain** | 0.113 | 0.103 | 4.385, <.001 | 1.889, 0.965 | 3.493, 0.014 | 0.383, 1 | |
| **USA** | 0.189 | 0.125 | 2.887, 0.084 | 0.390, 1 | 1.994, 0.767 | -1.115, 1 | -1.498, 1 |

Table 5. *Pairwise background comparisons for Japan.*

| Country | Mean | SD | t-statistic, p-value | | | |
|---|---|---|---|---|---|---|
| | | | Depicted | Amorphic | Empty | Backfix |
| **Depicted** | 0.532 | 0.088 | | | | |
| **Amorphic** | 0.056 | 0.033 | 14.832, <.001 | | | |
| **Empty** | 0.335 | 0.075 | 6.148, <.001 | -8.683, <.001 | | |
| **Backfix** | 0.061 | 0.078 | 14.682, <.001 | -0.150, 1 | 8.533, <.001 | |
| **Impossible** | 0.017 | 0.012 | 16.047, <.001 | 1.215, 1 | 9.898, <.001 | -1.365, 1 |

Table 6. *Pairwise background comparisons for China.*

| Country | Mean | SD | t-statistic, p-value | | | |
|---|---|---|---|---|---|---|
| | | | Depicted | Amorphic | Empty | Backfix |
| **Depicted** | 0.592 | 0.187 | | | | |
| **Amorphic** | 0.168 | 0.099 | 7.046, <.001 | | | |
| **Empty** | 0.208 | 0.163 | 6.380, <.001 | -0.665, 1 | | |
| **Backfix** | 0.012 | 0.022 | 9.650, <.001 | 2.605, 0.133 | 3.270, 0.024 | |
| **Impossible** | 0.019 | 0.024 | 9.519, <.001 | 2.474, 0.182 | 3.139, 0.034 | 0.131, 1 |

Table 7. *Pairwise background comparisons for Russia.*

| Country | Mean | SD | t-statistic, p-value | | | |
|---|---|---|---|---|---|---|
| | | | Depicted | Amorphic | Empty | Backfix |
| **Depicted** | 0.548 | 0.094 | | | | |
| **Amorphic** | 0.082 | 0.065 | 12.225, <.001 | | | |
| **Empty** | 0.29 | 0.111 | 6.772, <.001 | -5.453, <.001 | | |
| **Backfix** | 0.058 | 0.056 | 12.843, <.001 | 0.618, 1 | 6.071, <.001 | |
| **Impossible** | 0.023 | 0.023 | 13.765, <.001 | 1.539, 1 | 6.992, <.001 | -0.922, 1 |

Table 8. *Pairwise background comparisons for Nigeria.*

| Country | Mean | SD | t-statistic, p-value | | | |
|---|---|---|---|---|---|---|
| | | | Depicted | Amorphic | Empty | Backfix |
| **Depicted** | 0.702 | 0.102 | | | | |
| **Amorphic** | 0.063 | 0.049 | 15.450, <.001 | | | |
| **Empty** | 0.132 | 0.077 | 13.768, <.001 | -1.682, 1 | | |
| **Backfix** | 0.068 | 0.113 | 15.330, <.001 | -0.121, 1 | 1.561, 1 | |
| **Impossible** | 0.036 | 0.053 | 16.100, <.001 | 0.650, 1 | 2.332, 0.254 | -0.771, 1 |

Table 9. *Pairwise background comparisons for Spain.*

| Country | Mean | SD | t-statistic, p-value | | | |
|---|---|---|---|---|---|---|
| | | | Depicted | Amorphic | Empty | Backfix |
| **Depicted** | 0.811 | 0.128 | | | | |
| **Amorphic** | 0.042 | 0.028 | 20.174, <.001 | | | |
| **Empty** | 0.113 | 0.103 | 18.318, <.001 | -1.856, 0.717 | | |
| **Backfix** | 0.014 | 0.023 | 20.917, <.001 | 0.743, 1 | 2.599, 0.135 | |
| **Impossible** | 0.021 | 0.027 | 20.734, <.001 | 0.561, 1 | 2.417, 0.209 | 0.183, 1 |

Table 10. *Pairwise background comparisons for the United States.*

| Country | Mean | SD | t-statistic, p-value | | | |
|---|---|---|---|---|---|---|
| | | | Depicted | Amorphic | Empty | Backfix |
| **Depicted** | 0.734 | 0.192 | | | | |
| **Amorphic** | 0.04 | 0.045 | 13.154, <.001 | | | |
| **Empty** | 0.189 | 0.125 | 10.337, <.001 | -2.816, 0.078 | | |
| **Backfix** | 0.013 | 0.026 | 13.661, <.001 | 0.507, 1 | 3.324, 0.020 | |
| **Impossible** | 0.024 | 0.023 | 13.454, <.001 | 0.301, 1 | 3.117, 0.036 | 0.207, 1 |

Table 11. *Pairwise country comparisons for re-establishing length.*

| Country | Mean | SD | t-statistic, p-value | | | | |
|---|---|---|---|---|---|---|---|
| | | | Japan | China | Russia | Nigeria | Spain |
| **Japan** | 0.732 | 0.262 | | | | | |
| **China** | 0.377 | 0.355 | -2.479, 0.245 | | | | |
| **Russia** | 0.64 | 0.302 | 0.639, 1 | -1.840, 1 | | | |
| **Nigeria** | 0.351 | 0.276 | 2.657, 0.155 | 0.178, 1 | -2.018, 0.728 | | |
| **Spain** | 0.199 | 0.191 | 3.721, 0.007 | 1.242, 1 | 3.082, 0.049 | 1.064, 1 | |
| **USA** | 0.378 | 0.467 | 2.471, 0.250 | -0.007, 1 | 1.832, 1 | -0.186, 1 | -1.250, 1 |
